# Multiple Roles of TRIM21 in Virus Infection

**DOI:** 10.3390/ijms24021683

**Published:** 2023-01-14

**Authors:** Xue Li, Lin Yang, Si Chen, Jiawei Zheng, Huimin Zhang, Linzhu Ren

**Affiliations:** College of Animal Sciences, Key Lab for Zoonoses Research, Ministry of Education, Jilin University, 5333 Xi’an Road, Changchun 130062, China

**Keywords:** tripartite motif protein 21 (TRIM21), viruses, interaction

## Abstract

The tripartite motif protein 21 (TRIM21) belongs to the TRIM family, possessing an E3 ubiquitin ligase activity. Similar to other TRIMs, TRIM21 also contains three domains (named RBCC), including the Really Interesting New Gene (RING) domain, one or two B-Box domains (B-Box), and one PRY/SPRY domain. Notably, we found that the RING and B-Box domains are relatively more conservative than the PRY/SPRY domain, suggesting that TRIM21 of different species had similar functions. Recent results showed that TRIM21 participates in virus infection by directly interacting with viral proteins or modulating immune and inflammatory responses. TRIM21 also acts as a cytosol high-affinity antibody Fc receptor, binding to the antibody–virus complex and triggering an indirect antiviral antibody-dependent intracellular neutralization (ADIN). This paper focuses on the recent progress in the mechanism of TRIM21 during virus infection and the application prospects of TRIM21 on virus infection.

## 1. Introduction

The tripartite motif proteins (TRIMs) belong to the E3 ubiquitin ligase family, discovered in almost all multicellular animals [1,2,3]. TRIMs contain more than 80 members in humans and have diverse functions, which involve most life activities, including proliferation, differentiation, apoptosis, gene expression, signal transmission, damage repair, inflammation, and immunity [1,2,3,4,5,6,7].

The amino-terminal of TRIM protein contains three relatively conservative domains (RBCC), including the Really Interesting New Gene (RING) domain, B-Box domains (B-Box), and coiled-coil domain (CC) [1,3]. The RING domain is a zinc finger motif formed by cysteine, histidine, and Zn^+^, which confers TRIMs with E3 ubiquitin ligase activity [1,2,3]. The RING domain can mediate the ubiquitination of the target protein by binding with E2 ubiquitin ligase, thus changing the fate of the modified protein [1,2,3]. In addition, some TRIMs without the RING domain, such as porcine TRIM14 (pTRIM14), pTRIM16, and pTRIM29, may interact with other TRIMs as a scaffold protein, thus indirectly participating in the ubiquitination of target proteins [8]. The B-Box domains, including B-Box1 and/or B-Box2, also contain cysteine–histidine–zinc binding motifs [1,2,3]. The B-Box domains participate in the oligomerization of heterologous or homologous proteins, thus triggering the formation of macromolecules and their subcellular localization [1,2,3]. Furthermore, the B-Box also exhibits E3 ubiquitin ligase activity, which may make up for some TRIMs lacking RING. The CC domain of TRIM is a supercoiled domain composed of several α helices, which play a critical role in forming TRIM homologous polymers [1,3].

The C-terminal of TRIM involves protein–protein interaction due to its hypervariable region [1,2,3]. In addition, the C-terminal of most TRIMs contain PRY/SPRY domain, which can mediate the interaction between TRIMs and RNA or protein, and some of them have Fc receptor-like activity [1,2,3]. Numerous results showed that the E3 ubiquitin ligase activity and PRY/SPRY domain also participate in the cellular antiviral responses [3,9,10,11,12,13,14,15].

**Figure 1:**
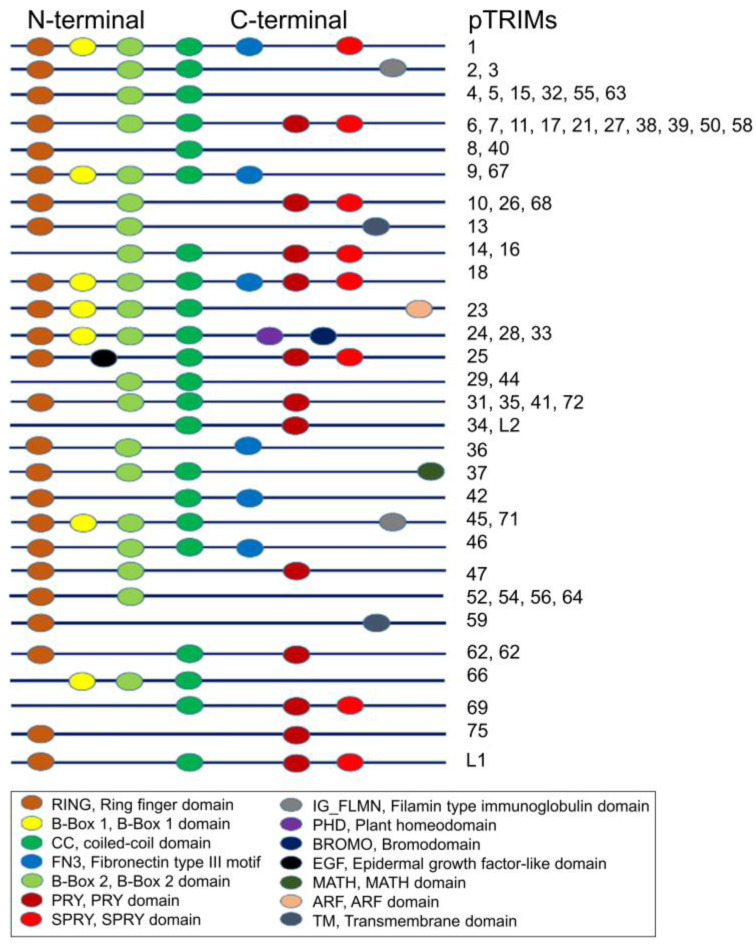
Predicated structures of porcine TRIMs. Sixty-one sequences of porcine TRIMs were analyzed, and the possible domains were predicated. The N-terminal of porcine TRIMs is conservative. In contrast, the C-terminal has a variety of variable domains. pTRIM, porcine TRIM.

TRIMs of the same species may be composed of distinct domains, whereas TRIMs are relatively conserved among different species [1,2,4,5,6,7,8,16,17]. For example, based on porcine TRIM sequences, we found that the N-terminal of porcine TRIMs is conservative (Figure 1). In contrast, the C-terminal has a variety of variable domains. It is worth noting that most porcine TRIMs have RBCC-PRY/SPRY domains, among which porcine TRIM6, 7, 11, 17, 21, 27, 38, 39, 50, and 58 contain similar motifs. These TRIMs have one RNIG motif, one B-Box motif, a coiled-coil domain in the N-terminal, and a PRY/SPRY domain in the C-terminal part.

TRIM21 (also named Ro52) is highly active and located along the microtubule network in the cytoplasm [18]. TRIM21 was first identified in autoimmune patients, acting as a self-antigen [19]. Later, it was found that TRIM21 plays a crucial role in regulating the signal pathway of type I IFN during virus infection [20]. Furthermore, TRIM21 also acts as a cytosol high-affinity antibody Fc receptor, binding to the antibody–virus complex and triggering an indirect antiviral antibody-dependent intracellular neutralization (ADIN) [10,14,21,22]. Therefore, TRIM21 provides effective antiviral protection and is a critical way to induce innate immune responses. In this review, we discuss the recent progress on the mechanism of TRIM21 during virus infection and the application prospects of TRIM21 during virus infection.

## 2. Sequence and Structural Characteristics of TRIM21

**Figure 2:**
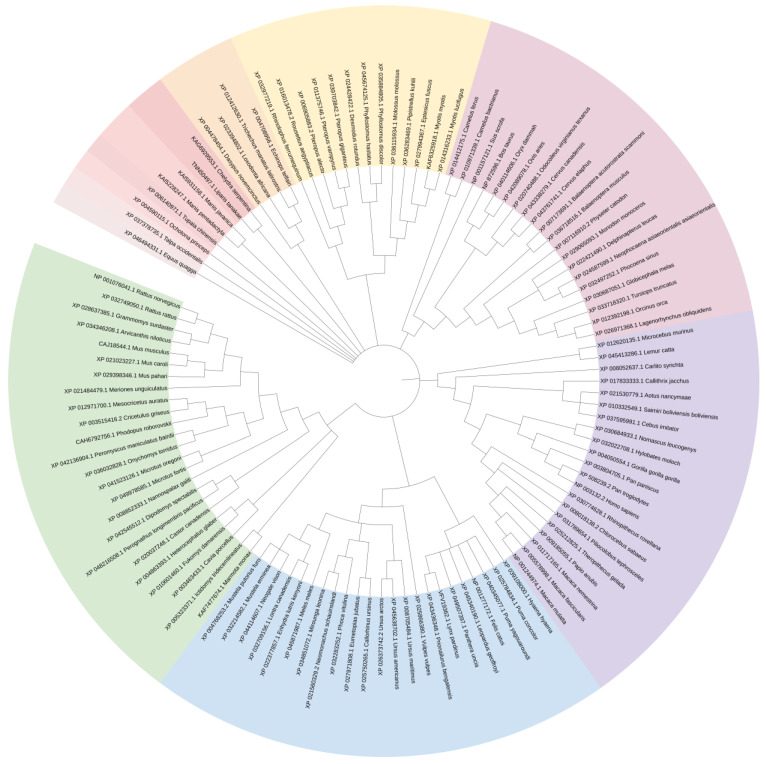
Genetic analysis of TRIM21 among different species. Amino acid sequences of porcine TRIM21 were compared with those of 112 other species using MEGA 11.

TRIM21, also known as Ro52 or SS-A, is widely expressed in various species [20,23,24]. We compared amino acid sequences of porcine TRIM21 with those of 112 other species (Supplemental Table S1) using MEGA 11 [25]. The results showed that porcine TRIM21 and TRIM21 derived from 19 other species were grouped into the same clade (Figure 2). The homology between porcine TRIM21 and TRIM21 of 19 species was more than 80% (Table 1), including *Balaenoptera musculus* (GenBank XP_036718516.1), *Balaenoptera acutorostrata scammoni* (XP_007173691.1), *Lagenorhynchus obliquidens* (XP_026971368.1), *Tursiops truncates* (*XP_033718320.1*), *Orcinus orca* (*XP_012392198.1*), *Globicephala melas* (*XP_030687051.1*), *Physeter catodon* (*XP_007116910.2*), *Cervus elaphus* (*XP_043761741.1*), *Cervus canadensis* (*XP_043338279.1*), and *Delphinapterus leucas* (*XP_022421490.1*), etc. Porcine TRIM21 has the highest homology with TRIM21 from *B. musculus* and *B. acutorostrata scammoni*, with 83.8%. However, the homology of porcine TRIM21 with TRIM21 from *Homo sapiens* and *Mus musculus* was 76.50 % and 66.02%, respectively.

**Figure 3:**
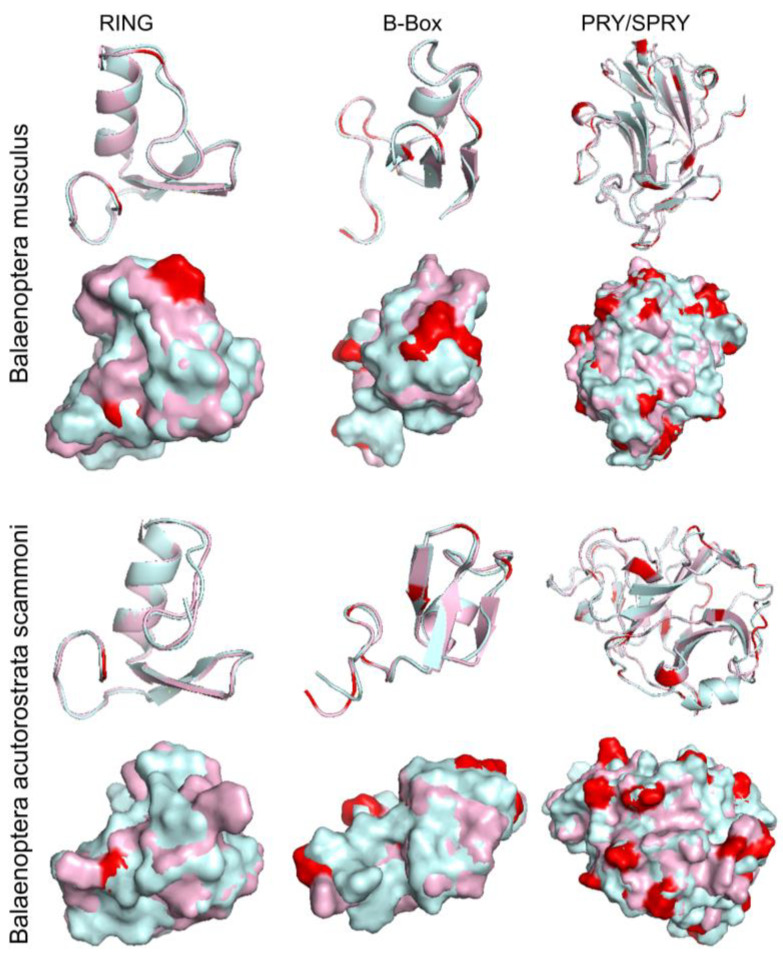
Predicated structural similarity between porcine TRIM21 and TRIM21 of *B. musculus* or *B. acutorostrata* scammony. Palecyan, porcine TRIM21; light pink, TRIM21 of *B. musculus* or *B. acutorostrata* scammony. Red indicates different residues of the RING and B-Box domains of TRIM21.

**Figure 4:**
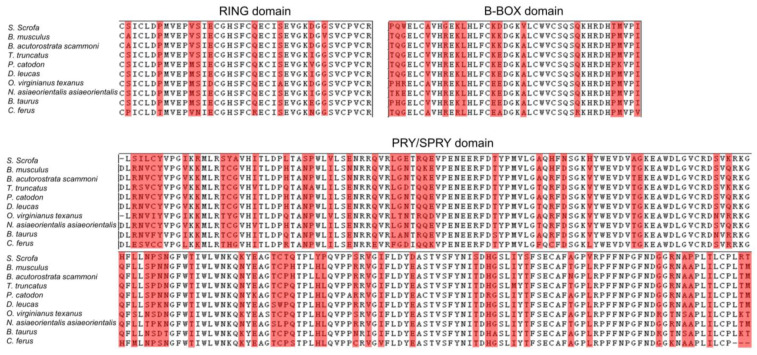
Comparison of TRIM21 derived from swine and eight other species. The conserved residue is labeled in white. Red indicates different amino acids.

The domain similarity of porcine TRIM21 protein and TRIM21 of the first species, *B. musculus* and *B. acutorostrata scammony*, were compared using the online program Pymol 2.0. As shown in Figure 3 and Figure 4, the similarity between the two TRIM proteins is relatively high, especially in the RING and B-Box domains. These results indicate that TRIM21 is more conservative in different species. Moreover, three motifs are conserved in TRIM21, two of which (motif 1: aa 403–452, motif 3: aa 339–388) locate in the SPRY domain (aa 339–466), and the third one (motif 2: aa 9–58) is in the RING domain (aa 16–54). The evolutionary conservation of these domains among species further suggests that TRIM21 has similar or identical functions in different species.

## 3. Potential mechanisms of TRIM21 in virus infection

### 3.1. Direct Interaction with Viral or Host Protein to Affect Virus Infection and Immune Responses

**Figure 5:**
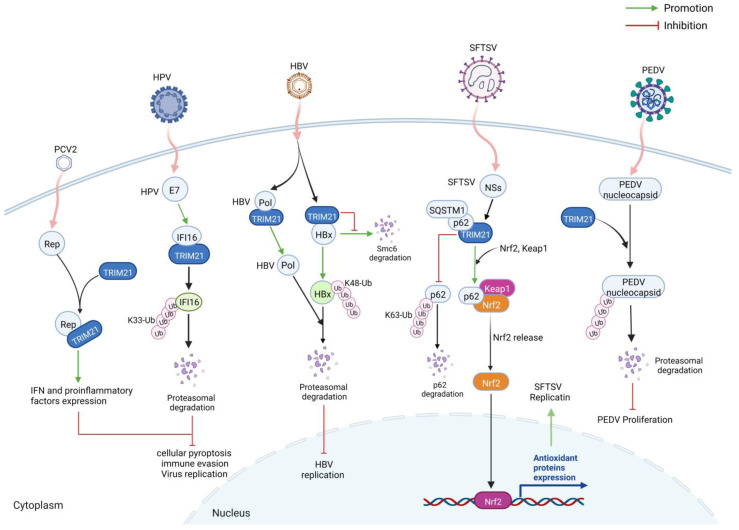
TRIM21 affects virus infection and immune responses by direct interacting with viral or other host proteins via its PRY/SPRY domain. Therefore, viral protein or host immune molecules are ubiquitinated by the RING domain of TRIM21 and then degraded by the proteasome, thus inhibiting virus infection. On the contrary, SFTSV can disturb the interaction between TRIM21 and p62, enhancing antioxidant responses and the p62-Keap1-Nrf2 pathway for SFTSV replication. Figure created with BioRender.com.

TRIMs can affect virus infection by directly participating in different stages of the virus infection cycle or targeting virus proteins (Figure 5). Generally, this interaction, especially the antiviral function of TRIM21, is related to the E3 ubiquitin ligase activity of its RING domain.

The interaction between the PRY/SPRY domain of TRIM21 and the virus protein narrowed the distance between the RING domain of TRIM21 and the viral protein. Then, the E3 ubiquitin ligase activity of the TRIM21 RING domain can directly ubiquitinate virus protein, thus promoting viral protein to be degraded by the proteasome pathway and inhibiting virus infection. As reported, TRIM21 exhibits antiviral activities on PRRSV infection via its E3 ubiquitin ligase of the RING domain [11]. Furthermore, TRIM21 inhibits hepatitis B virus (HBV) infection by interacting with the TP domain of HBV DNA polymerase (Pol) through the SPRY domain [9]. This interaction causes the degradation of the viral Pol via the ubiquitin–proteasome pathway induced by the RING domain of TRIM21 [9]. In addition, another group found that TRIM21 can inhibit HBV replication by directly triggering the K48-linked polyubiquitination, followed by proteasome degradation of viral HBx protein in the early step of the infection [10]. This interaction depends on both RING and PRY/SPRY domains of TRIM21, suppressing the HBx-dependent degradation of structural maintenance of chromosome 6 (Smc6) and inhibiting HBV replication [10].

Wang et al. found that TRIM21 directly ubiquitinates the nucleocapsid of the porcine epidemic diarrhea virus (PEDV), inhibiting PEDV proliferation [26]. Notably, PEDV infection downregulates endogenous expression of TRIM21 in African green monkey kidney cells (Vero cells) and porcine kidney cells (LLC-PK1 cells) [26]. However, another group reported that the expression of TRIM21 was upregulated after PEDV infection in porcine alveolar macrophages [27]. Therefore, the reason for the significant difference of TRIM21 expression in different cells during PEDV infection needs to be further clarified.

On the contrary, the interaction between TRIM21 and virus protein also can facilitate virus infection or immune escape for some viruses. For example, Song et al. reported that human papillomavirus (HPV) oncoprotein E7 can enhance the interaction between TRIM21 and interferon-gamma inducible protein 16 (IFI16) [28]. Further studies showed these interactions depend on the PRY/SPRY domain of TRIM21 and the PYD domain of IFI16 [28]. Thus, HPV E7 induced the K33-linked ubiquitination of IFI16 by interacting with the E3 ligase TRIM21, causing the ubiquitin–proteasome-mediated degradation of IFI16 [28]. The degradation of the IFI16 inflammasome leads to cellular pyroptosis and virus evasion from immune surveillance [28]. It is worth noting that TRIM21 can regulate redox reaction by directly binding to SQSTM1/p62 via the PRY/SPRY domain and mediate the K63-linked ubiquitylation of p62 at residue lysine (K)7 of the N-terminal PB1 domain [29]. After that, the oligomerization and sequestration function of p62 was abolished, leading to the formation of Kelch-like ECH-associated protein 1 (Keap1) and nuclear factor erythroid 2-related factor 2 (Nrf2) complex, followed by decreasing antioxidant response and enhancing cell death [29,30]. Choi et al. found that during severe fever with thrombocytopenia syndrome virus (SFTSV) infection, viral nonstructural proteins (NSs) interacted with the SPRY domain of TRIM21, thus eliminating the interaction between TRIM21 and p62 [31]. The free p62 binds to Keap1 and releases Nrf2 in the cytoplasm, which is then transferred into the nucleus and activates the transcription of target antioxidant genes, leading to antioxidant responses [29,30,31]. In addition, the activation of the p62-Keap1-Nrf2 pathway enhances the expression of the *Cd36* gene, thereby increasing CD36-mediated lipid uptake and providing a preferred environment for SFTSV replication [31]. The activation of the p62-Keap1-Nrf2 also contributes to viral pathogenesis [31]. In addition, we found that porcine TRIM21 was enhanced during porcine Circovirus 2 (PCV2) infection, which prompted PCV2 replication by decreasing cellular apoptosis and increasing interferons and proinflammatory factors [32]. Furthermore, the viral replicase can be co-immunoprecipitated with TRIM21 and confocal with TRIM21 in the cytoplasm (data not shown), suggesting an interaction between porcine TRIM21 and PCV2 replicase. Elucidating he exact mechanism involved is in progress. These results indicate a positive correlation between porcine TRIM21 and PCV2 infection.

Therefore, the direct interaction between viral proteins and TRIM21 may produce different or opposite results, inhibiting or favoring the virus’s life cycle, depending on the virus. These results suggest that TRIM21 is a promising target for therapeutic strategies against virus infection.

### 3.2. Regulating Immune and Inflammatory Responses

#### 3.2.1. TRIM21 Is a Negative Regulator of Immune Responses

**Figure 6:**
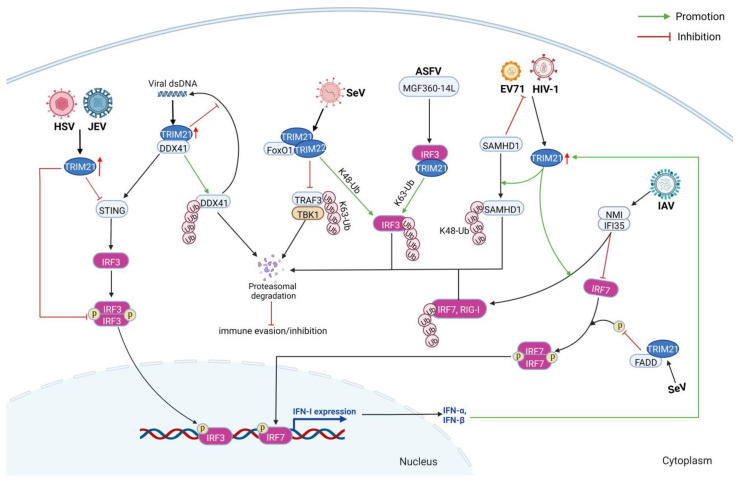
TRIM21 is a negative regulator of immune responses during virus infection. The ubiquitination of IRF3/7 or its upstream sensors by TRIM21, such as STING, DDX41, NMI/IFI35, and RIG-I, leads to immune suppression during virus infection. Finally, the replications of viruses, including SeV, IAV, HSV-1, ASFV, and JEV, were enhanced in TRIM21-dependent manners. Figure created with BioRender.com.

The immune response is a primary and effective antiviral strategy during virus infection. However, the virus can utilize various modes to regulate the immune responses to prompt its replication and proliferation. One crucial immune escape strategy for virus infection is the negative regulation of immune responses via TRIM21 and its E3 ubiquitin ligase activity (Figure 6) [33].

After sensing the pathogen, the E3 ubiquitin ligase activity of TRIM21 promotes the degradation of IFN regulatory factor 3 (IRF3), resulting in negative regulation of the production of IFN-β [12,34]. TRIM21 directly interacts with IRF3 through its SPRY domain, leading to polyubiquitination and proteasome degradation of IRF3 [34]. For example, the nonstructural protein MGF360-14L of African swine fever virus (ASFV) can interact with IRF3 and induce degradation of IRF3 via K63 ubiquitination mediated by TRIM21, thus suppressing the type I IFN response [35]. Furthermore, TRIM21 is upregulated in the epithelial keratitis mouse model during herpes simplex virus 1 (HSV-1) infection [36,37]. Highly expressed TRIM21 can suppress the type I IFN responses by inhibiting the stimulator of interferon genes (STING)/IRF3 signal pathway, thus enhancing HSV-1 infection in corneal epithelial cells [36]. Unexpectedly, levels of infectious HSV-1 were significantly higher in the trigeminal ganglia of TRIM21 deficient mice than that of the WT animals at seven days postinfection, with an elevation in *HSV-1 lytic* gene expression [37]. Highly expressed TRIM21 also improves the production of IL-6 and TNF-α, thus causing severe epithelial keratitis in the HSV-1 infection mouse model [36]. TRIM21 was upregulated in cells infected with the Japanese encephalitis virus (JEV) [12]. TRIM21 can inhibit phosphorylated IRF3 (p-IRF3) levels and thus suppress the level of IFN-β [12]. These results indicate that the direct modification of IRF3 by TRIM21 inhibits the IRF3 signaling pathway and IFN production.

Targeting upstream molecules of the IRF pathways by TRIM21, such as DEAD-box protein 41 (DDX41), STING, and RIG-I, is also a strategy to inhibit immune responses. DDX41 belongs to DExD/H-box RNA helicases, characterized by two conserved RecA-like domains [38]. DDX41 acts as an intracellular double-stranded DNA (dsDNA) sensor, which can trigger type I IFN responses via the signaling adaptor STING [39,40]. However, the interaction between TRIM21 and DDX41 contributes to immune suppression during virus infection. Zhang et al. found that the SPRY/PRY domain of TRIM21 interacts with the DEADc domain of DDX41, leading to the Lys48 (K48) ubiquitination at Lys9 and Lys115 of DDX41, followed by the degradation of DDX41 and immune suppression [39]. In addition, the interferon-inducible protein 35 (IFI35) and N-myc and STAT interacting protein (NMI) complex can interact with TRIM21 and induce a negative feedback inhibition on IFN-β production [41]. IFI35 mediates the proteasomal degradation of the RIG-I-like receptor (RLR), acting as a negative regulator of the RIG-I signaling pathway [41,42]. NMI is another interferon-inducible protein that can inhibit virus-induced type I IFN by triggering K48-linked ubiquitination and proteasomal degradation of IRF-7 [43,44]. As reported, TRIM21 induces K63-linked ubiquitination on Lys22 residue of NMI by interacting with the coiled-coil domain of NMI with its SPRY domain [44], which could activate NMI and enhance the formation and stability of the NMI/IFI35 complex. Meanwhile, the NMI/IFI35 domain 1 (NID1) and NID2 located at the carboxy-terminal of NMI can interact with IRF7 during influenza A virus (IAV) infection [44]. This interaction causes the ubiquitination and proteasomal degradation of IRF7 by recruiting TRIM21 [44]. Another factor in suppressing the RIG-I signaling pathway is the host transcription factor forkhead box O1 (FoxO1) [25]. During Sendai virus (SeV) infection, FoxO1 recruited TRIM22 or TRIM21 through its DNA binding domain (DBD) to promote K48-linked ubiquitination of IRF3, thus destroying IRF3 [25]. Besides, FoxO1 also inhibits the K63-linked ubiquitination of TRAF3, thus digesting the TRAF3/TBK1 complex [25].

TRIM protein can also target host protein, thus inhibiting immune activation and affecting virus infection. Sterile alpha motif and histidine-aspartic acid domain-containing protein 1 (SAMHD1) is a restriction factor of retroviruses and enterovirus 71 (EV71) [15,45]. Furthermore, EV71 infection upregulated the expressions of IFN-α and IFN-β, which were responsible for enhancing the TRIM21 expression in vitro and in vivo [15]. On the contrary, TRIM21 promotes the production of type I IFN in a SAMHD1-dependent manner [15]. Moreover, TRIM21 can directly interact with 1-547 aa of SAMHD1, especially residues G153 and G183, through its PRY and SPRY domains and then induce ubiquitination at K622 of SAMHD1 via K48 linkage ubiquitin [15]. Finally, SAMHD1 was degraded by proteasomes in a TRIM21-dependent manner, and the replication of EV71 was enhanced. The degradation of SAMHD1 also improves the infectivity of HIV-1 [15]. Meanwhile, TRIM21 interacts with the death-effector domain (DED) of the Fas-associated death domain (FADD) via its SPRY domain to enhance autoubiquitination of TRIM21 and the ubiquitination activity of TRIM21 on IRF7, but decrease the phosphorylation of IRF7 [46]. Furthermore, FADD and TRIM21 negatively synergistically regulate IRF7-mediated activation of IFN-α during SeV infection [46].

Therefore, the ubiquitination of IRF3/7 or its upstream sensors by TRIM21, such as STING, DDX41, NMI/IFI35, and RIG-I, leads to immune suppression during virus infection. Finally, the replications of viruses, including SeV, IAV, HSV-1, ASFV, and JEV, were enhanced in TRIM21-dependent manners.

#### 3.2.2. TRIM21 Can Positively Regulate Immune Responses

**Figure 7:**
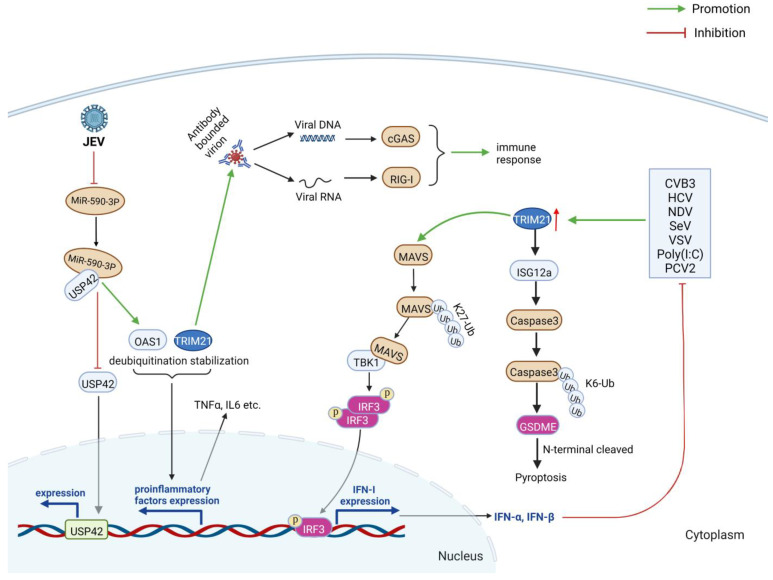
TRIM21 positively influences host immune responses during virus infection. TRIM21 has diverse activities during different virus infections, and its regulation of type I IFN and inflammatory responses are inconsistent during various virus infections. Figure created with BioRender.com.

Apart from negatively regulating immune responses, TRIM21 also enhances immune responses during some virus infections (Figure 7). For example, TRIM21 mediates K27-conjugated polyubiquitination of MAVS and up-regulates IRF3-induced type I IFN, thus promoting innate immune responses against RNA virus infection [20,47], suggesting TRIM21 is also a positive regulator of immune responses. Liu et al. evaluated the effect of TRIM21 on type I IFN production during Coxsackievirus B3 (CVB3) infection in vitro and in vivo [47]. They found that TRIM21 was upregulated during CVB3 infection, and the levels of type I IFN were positively related to that of TRIM21. Further studies demonstrated that the RING and PRY/SPRY domains of TRIM21 interact and promote the K27-linked ubiquitination of MAVS. After that, the IRF3 was phosphorylated and dimerized, followed by the induction of the type I IFN responses [47]. Thereby, CVB3 replication was inhibited, and cardiac inflammatory cytokines, pancreatic injury, and viral myocarditis were relieved. These results were further confirmed by other RNA viruses, including Newcastle disease virus (NDV), hepatitis C virus (HCV), vesicular stomatitis virus (VSV), Sendai virus (SeV), as well as nucleic acid mimic poly(I•C) [20,48]. During the infections of the above viruses, TRIM21 triggers the K27-linked polyubiquitination of MAVS, which leads to the interaction between TBK1 and MAVS, followed by enhancement of the IRF3-associated immune responses against virus infection [20,48]. The increase in TRIM21 during NDV infection induces the K6-linked polyubiquitination of ISG12a at lys69 residue through the interaction between the DII of ISG12a and the PRY/SPRY domain of TRIM21 [49]. The ubiquitinated ISG12a translocated into mitochondria and activated caspase 3, followed by cleavage of gasdermin-E (GSDME) and induction of cell pyroptosis by the N-terminal of GSDME [49].

TRIM21 induces the destruction of the antibody-bounded virion, followed by the recognition of the exposed viral genome in the cytoplasm by DNA or RNA sensors, such as cGAS or RIG-I, and the immune responses are activated [3,50]. In addition, cellular miR-590-3p can inhibit the expression of USP42 (ubiquitin specific peptidase 42) by directly interacting with the protein [51]. However, the miR-590-3p was downregulated during JEV infection, resulting in the upregulation of USP42 [51]. USP42 enhances the deubiquitination and stabilization of oligoadenylate synthetase 1 (OAS1) and TRIM21, leading to neuroinflammation and antiviral responses [51].

The level of TRIM21 in CD16^−^ monocytes was significantly lower than that of CD16^+^ cells and monocytes [52]. On the other hand, the level of TRIM21 was about six times higher in dendritic cells (DCs) and about 16 times more monocyte-derived macrophages (MDM) (*p* < 0.01) than that in monocytes [52]. These results indicate that TRIM21 is associated with proinflammatory cytokine production in CD16^+^ monocytes and macrophages during acute or chronic infections of enveloped viruses. We previously found that TRIM21 triggers immune and inflammatory responses by enhancing IFNs and proinflammatory factors, such as IL-6, IFN-β, IFN-γ, and TNF-α, in PCV2-infected cells [32].

Therefore, TRIM21 has diverse activities during different virus infections, and its regulation of type I IFN and inflammatory reactions are inconsistent during various virus infections.

### 3.3. Serving as a Cytosolic Fc Receptor

**Figure 8:**
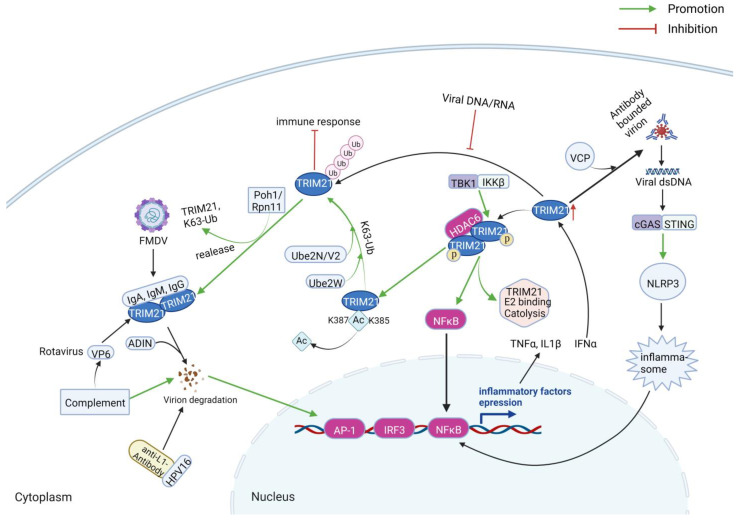
TRIM21 serves as a cytosolic Fc receptor. TRIM21 is a high-affinity cytosolic IgG receptor, which can bind with the antibody–virus complex and induce an indirect antiviral antibody-dependent intracellular neutralization (ADIN) and virion degradation via the ubiquitin–proteasome pathway. Figure created with BioRender.com.

TRIM21 also serves as a cytosolic IgG receptor, which can efficiently bind with the antibody–virus complex and induce an indirect antibody-dependent intracellular neutralization (ADIN) to degrade virion via the ubiquitin–proteasome pathway (Figure 8) [10,14,21,53,54,55,56,57]. However, although monomer TRIM21 is a highly active E3 ligase, the B-Box of TRIM21 combines with the E2 binding site of the RING domain, acting as an E2 mimic via electrostatic interaction and a salt bridge between residues R118 and E12, leading to inhibition of TRIM21 ubiquitination and preventing immune activation [58]. On the contrary, kinases IKKβ or TBK1 directly phosphorylate TRIM21 at residue S80 in the LxxIS motif of the RING domain to prevent B-Box inhibition, enhancing E2 binding, RING catalysis, NF-κB activation, and cytokine expression after DNA or RNA viruses’ infection [58]. During this process, catalytically inactive TRIM21 dimer binds with the target protein, such as antibody-bound virions or other target proteins [55]. Then, multiple TRIM21 molecules were recruited to the target, leading to RING dimerization and TRIM21 activation [55]. It is worth noting that the dimerization of TRIM21 is regulated by histone deacetylase 6 (HDAC6) [59]. HDAC6 binds to the PRY/SPRY motif of TRIM21 and deacetylates TRIM21 at residues lysine 385 and lysine 387 [59]. After that, TRIM21 catalyzes a mono-ubiquitin onto its N-terminus via the E2 enzyme Ube2W, followed by the K63-ubiquitin (Ub) chain extension via Ube2N/V2 [55,60,61]. Subsequently, the TRIM21 and target immune complex was degraded by the proteasome. Proteasome-associated deubiquitinase Poh1/Rpn11 participates in the deubiquitination of TRIM21, resulting in the release of TRIM21 and K63-polyubiquitin [60,61].

As reported, porcine TRIM21 was triggered by type I IFN during foot-and-mouth disease virus (FMDV) infection [14]. The complex formed by FMDV and FMDV-specific antibodies can be recognized by the Fc receptor domain of porcine TRIM21, which leads to the degradation of FMDV via ADIN [14]. Specific IgGs of the middle capsid VP6 of rotavirus can induce effective neutralization against the virus and protect the infected animal [62]. Nonetheless, the human papillomavirus (HPV16) pseudovirus bound with anti-L1 antibodies can be degraded intracellularly via the TRIM21-mediated proteasomal pathway [63]. The RING and PRY/SPRY domains are necessary for TRIM21-mediated degradation [14,64,65]. Moreover, the inhibition triggered by TRIM21 can synergize with complement-mediated inhibition of viral infection [66].

Moreover, TRIM21-mediated protein degradation also activates AP-1, NF-κB, and IRF signaling pathways, thus promoting the production of inflammatory cytokines and enhancing the antiviral activities of cells [14,60,61,67,68]. Meanwhile, the antiviral function of TRIM21 can be improved by IFN-α [57]. Immunoprotection can be induced by the cytosolic Fc receptor and E3 ubiquitin ligase activities of TRIM21 by targeting the nucleoprotein–antibody complex of lymphocytic choriomeningitis virus (LCMV) [69]. The non-neutralizing anti-nucleoprotein antibody derived from proteasome activates cytotoxic T cells (CTLs) and induces CTL responses against LCMV infection [69]. Zhang et al. found that the cell-penetrating whole-molecule monoclonal antibody targeting intracellular HBx can stimulate TRIM21-mediated ADIN and activate innate antiviral pathways, contributing to antibody-mediated HBV suppression in vitro and in vivo [67]. Notably, TRIM21-induced immune response is more sensitive to antibody affinity than ADIN [70], indicating that antibody-mediated ADIN is more prone to be stimulated than immune responses. In addition, TRIM21 can detect and bind the Fc fragment of IgA, IgG, and IgM in response to viral and bacterial pathogens [68,71], confirming that TRIM21 triggers antiviral inflammatory and immune signaling.

Antibody-coated viruses can stimulate phagocytosis, activate the formation of NLRP3-associated inflammasome, and release IL-1β [72]. Unexpectedly, part of antibody-coated viruses can escape into the cytosol. However, TRIM21 can detect the antibody-bounded virions in the cytosol, which then activate the inflammasome via the cGAS/STING-induced NLRP3 pathway and stimulate the NFκB pathway, followed by the expression of TNF and IL-1β [72]. During this process, ATPase p97/valosin-containing protein (VCP), an enzyme with segregase and unfoldase activity, plays a critical role in interacting with proteasomes [54,73]. TRIM21 recruits VCP and proteasome to destroy the virion–antibody complex and releases the viral genomes. Then, nucleic acid sensors, such as STING, MAVS, and RIG-I, detect the viral genome and induce immune signals in response to virus infection.

## 4. Application Prospects of TRIM21

Since TRIM21 possesses Fc receptor and E3 ubiquitin ligase activities, numerous groups have tried to develop virus infection detection and prevention methods based on TRIM21.

The first application based on TRIM21 is TRIM-AWAY, designed to degrade target protein or inhibit virus propagation via a proteasomal pathway [55,74]. Intracellular antibody neutralization mediated by the cytosolic IgG receptor of TRIM21 enables TRIM-AWAY to be promising for controlling and preventing virus infection. As reported, anti-L1 antibody opsonized HPV16 pseudovirus can induce the degradation of the pseudovirus by the TRIM21-mediated proteasome pathway [63], indicating that HPV16 therapy is feasible by TRIM-AWAY. Furthermore, TRIM21-cyclophilin A (TRIM21CypA) fusion proteins expressed by lentiviral vector inhibited the proliferation of replication-competent HIV-1 in human cells [75]. T cells treated with TRIM21CypA showed preferential survival against wild-type HIV [75]. Notably, TRIM21CypA-mediated inhibition did not affect the endogenous TRIM5α and TRIM21-mediated antiviral activities [75]. However, the low tissue penetration of full-size antibodies may limit the intracellular neutralization of the target protein. Therefore, Chen et al. developed a TRIMbody-AWAY technique by fusing the nanobody and RBCC motif of TRIM21 [74]. The TRIMbody induces efficient intracellular degradation of the target protein [74]. Moreover, TRIM21 simultaneously enhances immune signaling and stimulates CD8 T cell responses [76]. Unexpectedly, TRIM21-mediated neutralization blocks the expression of the transgene in the adenoviral vector due to pre-existing antibodies against adenoviral [76]. TRIM21 also decreases cytotoxic T cells induced by adenovirus-based vaccine and prevents the protective effect against subsequent virus infection [76]. Therefore, proper immunization, such as intranasal administration and lower viral dose, may circumvent problems caused by the interaction between TRIM21 and preexisting antibodies [76].

TRIM21-based virus protein or antibody detection is also promising. Albecka et al. developed an electroporated antibody-dependent neutralization assay (EDNA) based on the TRIM21-mediated intracellular neutralization to detect nucleoprotein antibodies of coronaviruses, including murine hepatitis virus (MHV) and SARS-CoV-2 [77]. The EDNA can measure nucleoprotein antibodies in SARS-CoV-2 convalescents, which were widely used to diagnose previous infections with SARS-CoV-2. SARS-CoV-2 convalescent patients produce robust neutralizing N antibodies related to the number of active N-specific T cells [77]. These results indicate that the EDNA can be used to evaluate SARS-CoV-2 infection and immune signals induced by the nucleoprotein-based vaccine.

## 5. Conclusions and Perspectives

In summary, TRIM21 affects virus infection by directly regulating virus proliferation and modulating immune and inflammatory responses. However, TRIM21 affects virus infection in a virus-dependent manner. The replications of viruses, including ASFV, EV71, HPV, HSV-1, IAV, JEV, PCV2, and SeV, were enhanced by TRIM21, while TRIM21 inhibits the infections of other viruses, such as CVB3, FMDV, HBV, HCV, PEDV, and VSV, etc. The critical domains of TRIM21 are PRY/SPRY and RING, which recognize the target protein and mediate the ubiquitination of the protein, respectively. The RING domain is conserved among species and retains E3 ubiquitin ligase activity. However, the PRY/SPRY domain is not very conservative, suggesting that it can interact with various proteins and has a multitargeting characteristic. Therefore, TRIM21 is a promising target for targeted therapy or prevention strategies against virus infection.

## Figures and Tables

**Table 1:** The homology between porcine TRIM21 and TRIM21 of 19 species.

GenBank No.	Species	Homology
XP_036718516.1	*Balaenoptera musculus*	83.80%
XP_007173691.1	*Balaenoptera acutorostrata scammoni*	83.80%
XP_026971368.1	*Lagenorhynchus obliquidens*	83.37%
XP_033718320.1	*Tursiops truncatus*	83.37%
XP_012392198.1	*Orcinus orca*	83.16%
XP_030687051.1	*Globicephala melas*	83.16%
XP_007116910.2	*Physeter catodon*	83.12%
XP_022421490.1	*Delphinapterus leucas*	82.94%
XP_043761741.1	*Cervus elaphus*	82.94%
XP_043338279.1	*Cervus canadensis*	82.94%
XP_032497252.1	*Phocoena sinus*	82.73%
XP_029065093.1	*Monodon monoceros*	82.73%
XP_020740488.1	*Odocoileus virginianus texanus*	82.30%
XP_024587599.1	*Neophocaena asiaeorientalis asiaeorientalis*	82.30%
NP_872596.1	*Bos taurus*	81.88%
XP_042089078.1	*Ovis aries*	81.72%
XP_014412175.1	*Camelus ferus*	81.47%
XP_010971339.1	*Camelus bactrianus*	81.47%
XP_040114606.1	*Oryx dammah*	81.02%

## Data Availability

All data generated or analyzed during this study are included in this published article and its Supplemental file.

## Supplementary Materials

The following supporting information can be downloaded at https://www.mdpi.com/article/10.3390/ijms24021683/s1.
